# Increased Platelet Reactivity in Idiopathic Pulmonary Fibrosis Is Mediated by a Plasma Factor

**DOI:** 10.1371/journal.pone.0111347

**Published:** 2014-10-22

**Authors:** Michael G. Crooks, Ahmed Fahim, Khalid M. Naseem, Alyn H. Morice, Simon P. Hart

**Affiliations:** 1 Centre for Cardiovascular and Metabolic Research, Hull York Medical School, Cottingham, United Kingdom; 2 Respiratory Medicine, New Cross Hospital, Wolverhampton, United Kingdom; Medical University of South Carolina, United States of America

## Abstract

**Introduction:**

Idiopathic Pulmonary Fibrosis (IPF) is a progressive, incurable fibrotic interstitial lung disease with a prognosis worse than many cancers. Its pathogenesis is poorly understood. Activated platelets can release pro-fibrotic mediators that have the potential to contribute to lung fibrosis. We determine platelet reactivity in subjects with IPF compared to age-matched controls.

**Methods:**

Whole blood flow cytometry was used to measure platelet-monocyte aggregate formation, platelet P-selectin expression and platelet fibrinogen binding at basal levels and following stimulation with platelet agonists. A plasma swap approach was used to assess the effect of IPF plasma on control platelets.

**Results:**

Subjects with IPF showed greater platelet reactivity than controls. Platelet P-selectin expression was significantly greater in IPF patients than controls following stimulation with 0.1 µM ADP (1.9% positive ±0.5 (mean ± SEM) versus 0.7%±0.1; p = 0.03), 1 µM ADP (9.8%±1.3 versus 3.3%±0.8; p<0.01) and 10 µM ADP (41.3%±4.2 versus 22.5%±2.6; p<0.01). Platelet fibrinogen binding was also increased, and platelet activation resulted in increased platelet-monocyte aggregate formation in IPF patients. Re-suspension of control platelets in plasma taken from subjects with IPF resulted in increased platelet activation compared to control plasma.

**Conclusions:**

IPF patients exhibit increased platelet reactivity compared with controls. This hyperactivity may result from the plasma environment since control platelets exhibit increased activation when exposed to IPF plasma.

## Introduction

Idiopathic pulmonary fibrosis (IPF) is the most common fibrotic interstitial lung disease and has a prognosis worse than many cancers [1]. Despite the significant morbidity and mortality associated with IPF its pathogenesis remains poorly understood and there is no curative treatment [2].

Epidemiological studies have demonstrated an association between IPF and vascular diseases including cardiovascular disease and venous thromboembolism [3], [4]. Local imbalance in the coagulation system has been demonstrated within the alveoli of IPF patients [5], [6] but the systemic vascular effects remain unexplained. Therapies targeting the coagulation cascade have been investigated in IPF, but a large randomised controlled trial of the vitamin K antagonist warfarin was stopped early because of increased mortality associated with the intervention [7]. This demonstrates that selectively targeting the coagulation system is ineffective and potentially harmful in IPF and suggests an alternative pathway may be responsible for the observed link between fibrosis and vascular disease.

Blood platelets play a central role in thrombosis through rapid activation and aggregation at sites of vascular injury. Transient activation of platelets also induces pro-inflammatory and pro-fibrotic effects [8] through the release of potent vasoactive mediators, inflammatory cytokines and pro-fibrotic factors from dense and α-granules. The role of platelets in lung disease is unclear, although increased platelet activation has been demonstrated in adult respiratory distress syndrome (ARDS) [8] and chronic obstructive pulmonary disease (COPD) [9]. While the role of platelets in IPF is unknown, platelet trapping in the lungs of mice following intravenous bleomycin administration strongly correlated with subsequent collagen deposition, suggesting a role in fibrogenesis in this animal model [10].

In the present study we measured markers of platelet activation in IPF patients under basal conditions and following stimulation with platelet agonists and investigated the effect of plasma from IPF patients on platelet activation. Our data demonstrate that IPF is associated with platelet hyperactivity that may be caused by the plasma environment.

## Methods

### Patient Selection

Patients with a diagnosis of idiopathic pulmonary fibrosis according to ATS/ERS criteria [11] were recruited from the interstitial lung disease clinic at a large teaching hospital. Age and sex matched controls without interstitial lung disease were attending out-patient clinics or were stable in-patients awaiting discharge. All patients were clinically stable and free from exacerbation or signs of infection at the time of sampling. A total of 20 IPF patients and 19 controls were recruited (table 1). Platelet activation and reactivity was assessed in 13 IPF patients and 12 controls giving a power of 0.8 with α of 0.05 to detect an absolute 5% difference in platelet-monocyte aggregate formation following stimulation with 1 µM ADP. The effects of IPF plasma on platelet activation were studied in a further 7 IPF patients and 7 controls. Additional information on the baseline characteristics of the participants is available as a data supplement (table S1 and S2 in file S1).

**Table 1:** Baseline characteristics of study participants.

Demographic	IPF (%)	Controls (%)
Number	n = 20	n = 19
Age (mean)	71.2	65.6
**Gender**		
Male	15 (75.0)	12 (63.2)
Female	5 (25.0)	7 (36.8)
**Comorbidities**		
COPD	2 (10.0)	8 (42.1)
Prev. malignancy	2 (10.0)	1 (5.3)
Hypertension	5 (25.0)	3 (15.8)
Diabetes mellitus	1 (5.0)	0 (0)
Ischaemic heart disease	1 (5.0)	0 (0)
TIA	4 (20.0)	0 (0)
Stroke	0 (0)	0 (0)
Atrial fibrillation	1 (5.0)	2 (10.5)
Chronic kidney disease	0 (0)	0 (0)
**Smoking Status**		
Non-smoker	3 (15.0)	8 (42.1)
Ex-smoker	14 (70.0)	8 (42.1)
Current smoker	2 (10.0)	2 (10.5)
Unknown	1 (5.0)	1 (5.3)
**Blood Counts – mean (SD)**		
Haemoglobin (g/dL)	14.2 (1.4)	12.9 (1.9)
White Cell Count (x10^9^/L)	7.4 (1.4)	6.9 (1.8)
Platelets (x10^9^/L)	241.6 (58.5)	267.4 (74.1)

COPD: chronic obstructive pulmonary disease; TIA: transient ischaemic attack. Blood count results were available for 20 IPF patients and 13 controls.

### Ethics Statement

Written informed consent was obtained from all participants and the study was approved by the Hull and East Riding Local Research Ethics Committee (LREC ref 08/H1304/54).

### Blood sampling and flow cytometric analysis of platelets

Venous blood was collected using a 21G butterfly needle and vacutainer system (Becton Dickinson Vacutainer Systems, UK). The first 4.5 mls of blood collected was discarded because artefactual platelet activation may occur during venepuncture. Citrated whole blood was added to tubes containing a saturating concentration of one or more of the following antibodies: fluorescein isothiocyanate (FITC)-conjugated monoclonal mouse anti-human CD42b (catalogue number 555472, BD Biosciences, UK); phycoerythrin (PE)-conjugated monoclonal mouse anti-human CD14 (product code MCA1568PE, AbD Serotec, UK); PE-conjugated monoclonal mouse anti-human CD62P (product code 304906, Biolegend, San Diego, California); or FITC-conjugated polyclonal rabbit anti-human fibrinogen (product code F0111, Dako, UK). To study the sensitivity of platelets to activation, some tubes were supplemented with adenosine diphosphate (ADP) (0.1, 1 and 10 µM) or the protease activated receptor - 1 (PAR1) agonist TFLLR (1, 5 and 10 µM). Single colour flow cytometry was used to assess platelet P-selectin expression and fibrinogen binding. Two-colour flow cytometry was used to identify the proportion of monocytes forming aggregates with one or more platelets (dual CD42b/CD14 positive) based on established methods [12].

### Effect of IPF Plasma on Control Platelets: Plasma Swap

In order to assess the effect of IPF plasma on control platelets a plasma swap approach was used. Blood from IPF patients and controls was used for the preparation of platelet-poor plasma. Washed platelets from control subjects were suspended in phosphate buffered saline (PBS) and divided into three aliquots. Plasma from the same donor was added to the first aliquot to form an autologous control. Plasma collected from a different control patient was added to the second aliquot to form an allogeneic control. Plasma from a patient with IPF was added to the third aliquot. The ratio of platelet suspension to plasma was 1∶1 (v/v). Samples were incubated with PE-conjugated anti-human CD62P antibody (product code 304906, Biolegend, San Diego, California) under basal conditions and in the presence of ADP (0.1, 1 and 10 µM) for 20 minutes prior to fixation and flow cytometric analysis. Additional detail on the methods for assessing the effects of IPF plasma on control platelets is provided in data supplement file S1.

### Data Analysis

Flow cytometry was performed using a FACS Calibur flow cytometer (Becton Dickinson, Oxford, UK) and the data were analysed using CellQuest Pro software (Becton Dickinson, Oxford, UK). Statistical comparisons of platelet-monocyte aggregate formation, platelet P-selectin expression and platelet fibrinogen binding between IPF and controls were performed using unpaired t-tests. A paired t-test was used to assess the difference in P-selectin expression between the groups in the plasma swap experiment. A p value of <0.05 was considered significant.

## Results

### Platelet-monocyte aggregate formation

**Figure 1:**
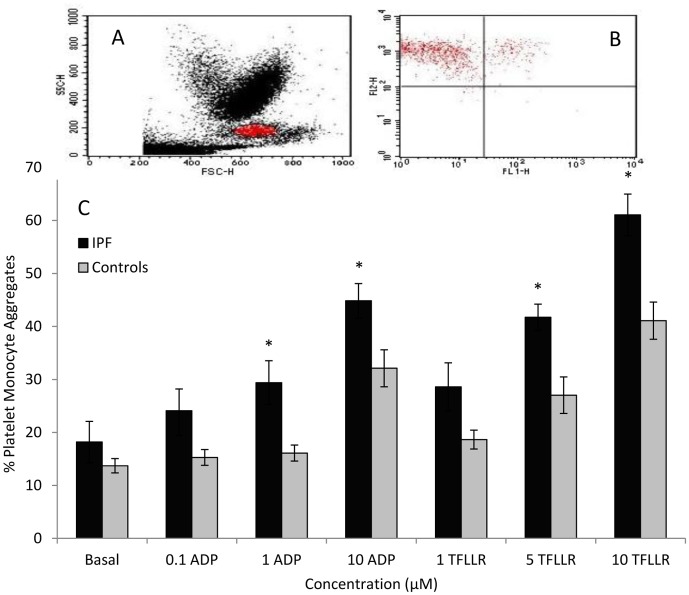
Platelet-monocyte aggregate formation in IPF patients and controls. A. Scatter plot demonstrating the different cell populations on whole blood flow cytometry, gated on monocytes. B. Quadrant plot demonstrating platelet-monocyte aggregates in the right upper quadrant. X-axis (FL1) shows CD42b staining and y-axis (FL2) shows CD14 staining. C. Percentage of monocytes forming aggregates with platelets at basal levels and in response to the platelet agonists ADP (0.1–10 µM) or TFLLR (1–10 µM). * P≤0.01.

**Figure 2:**
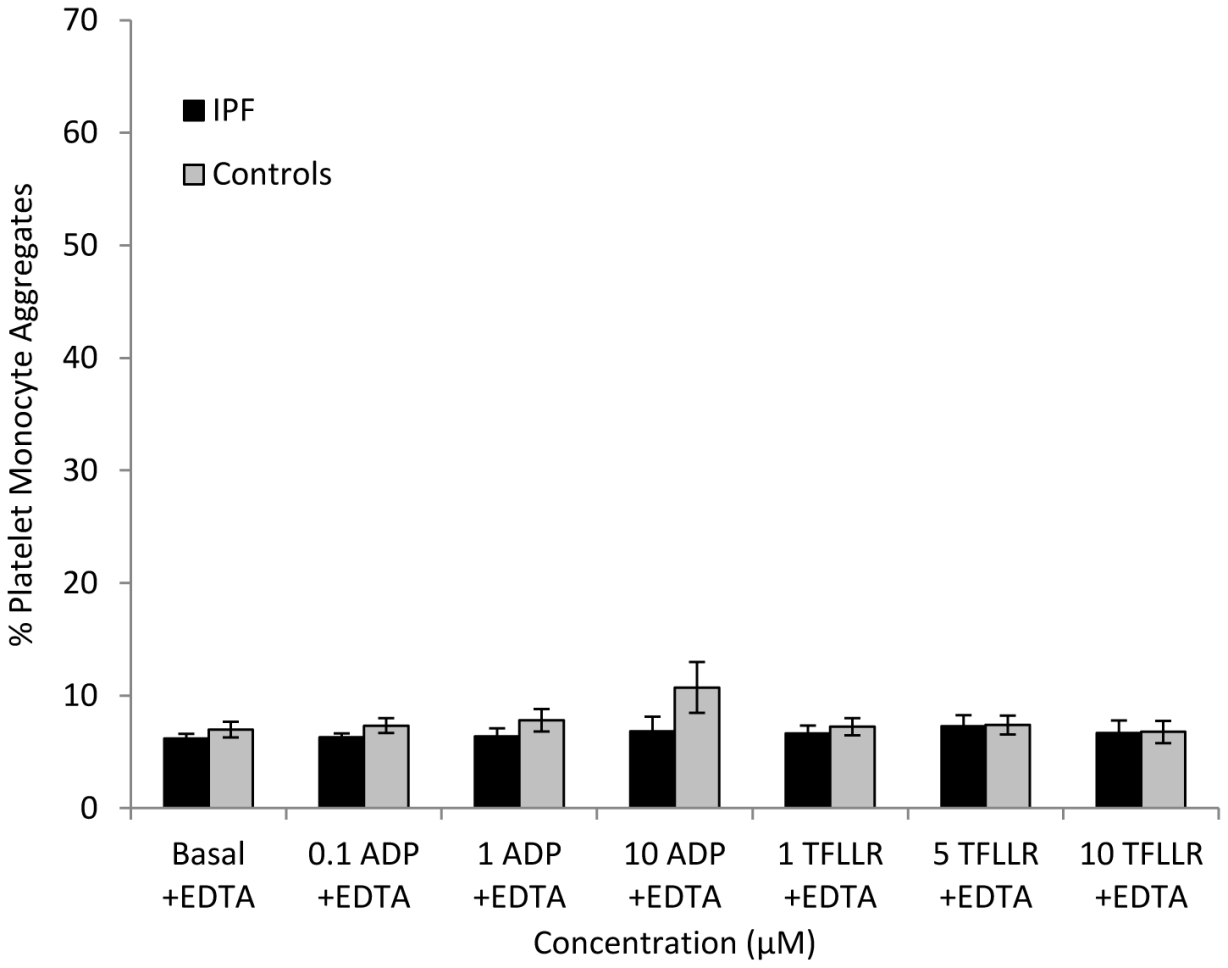
Percentage of monocytes forming aggregates with platelets in the presence of 1 mM EDTA under basal conditions and when stimulated with ADP (0.1–10 µM) or TFLLR (1–10 µM) in subjects with IPF and controls.

Patients with IPF and controls were matched for age and gender. When whole blood was stimulated with ADP (1–10 µM) or TFLLR (1–10 µM), subjects with IPF demonstrated significantly greater platelet-monocyte aggregate formation compared with controls (Figure 1 and table S3 in file S1). The percentage of monocytes with bound platelets was significantly greater in IPF patients following stimulation with 1 µM ADP (29.4%±4.1 (mean ± SEM) versus 16.1%±1.5 in controls; p = <0.01) and 10 µM ADP (44.8%±3.3 versus 32.1%±3.5; p = 0.01). Platelet-monocyte aggregate formation in blood from IPF patients was also significantly increased following stimulation with 5 µM and 10 µM TFLLR (figure 1) when compared to controls. Under basal (unstimulated) conditions, blood from IPF patients exhibited greater platelet-monocyte aggregate formation than controls but this did not reach statistical significance (18.2%±3.9 versus 13.7%±1.4; p = 0.3). The calcium-dependent nature of the interaction between platelets and monocytes was confirmed by addition of EDTA, which resulted in a marked reduction in aggregate formation that did not increase in response to agonist stimulation and did not vary significantly between the groups (figure 2).

### Platelet P-selectin expression and fibrinogen binding

**Figure 3:**
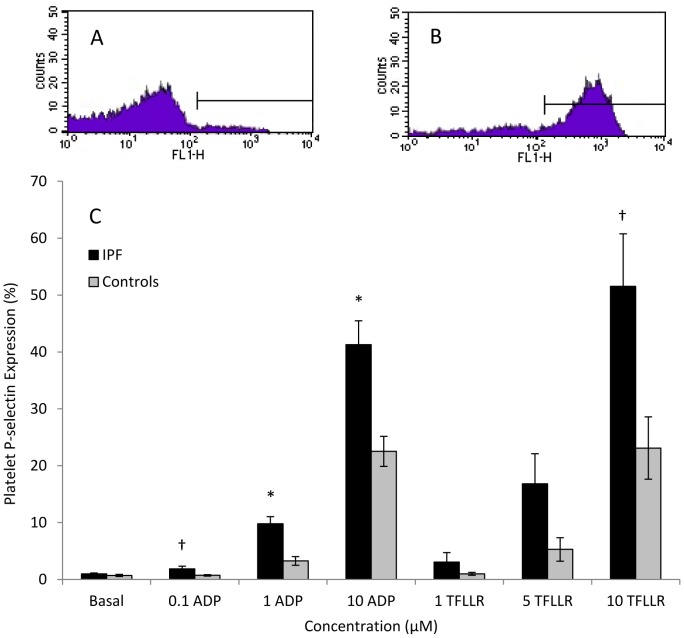
Platelet P-selectin expression in IPF patients and controls. A. Representative flow cytometry histogram demonstrating platelet P-selectin expression under basal (unstimulated) conditions. B. Flow cytometry histogram demonstrating platelet P-selectin expression following stimulation with 10 µM ADP. C. Percentage of platelets expressing P-selectin at basal levels and in response to the platelet agonists ADP (0.1–10 µM) or TFLLR (1–10 µM). † P<0.05, * P≤0.01.

Having established that platelet-monocyte aggregates were increased in IPF, we next examined other markers of platelet activation. Consistent with increased platelet-monocyte aggregates we found that platelet P-selectin expression was elevated in whole blood from IPF patients compared with controls. Stimulation with all concentrations of ADP led to greater increases in platelet P-selectin expression in IPF patients compared with controls (ADP 0.1 µM: 1.9%±0.5 positive platelets versus 0.7%±0.1; p = 0.03; ADP 1 µM: 9.8%±1.3 versus 3.3%±0.8; p = <0.01; ADP 10 µM: 41.3%±4.2 versus 22.5%±2.6; p = <0.01). Similar findings were observed following stimulation with TFLLR (figure 3 and table S4 in file S1). No differences were observed under basal (unstimulated) conditions.

**Figure 4:**
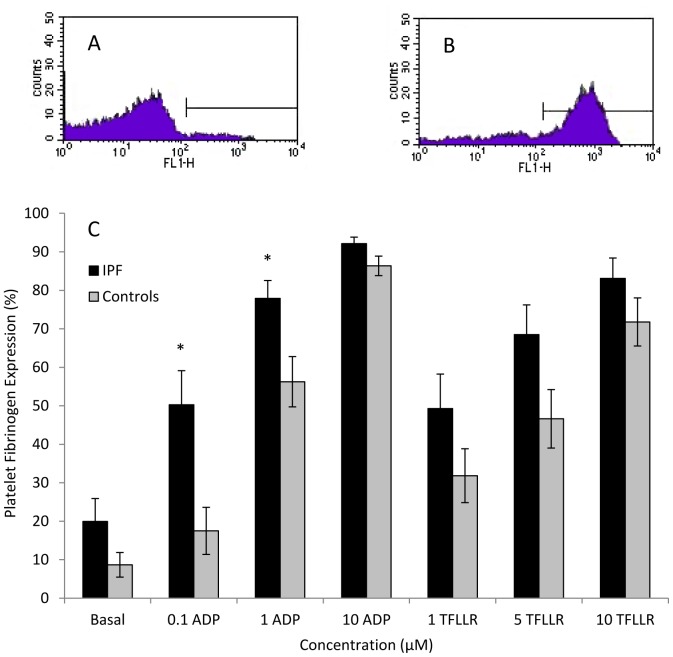
Platelet fibrinogen binding in IPF patients and controls. A. Representative flow cytometry histogram demonstrating platelet fibrinogen binding under basal (unstimulated) conditions. B. Flow cytometry histogram demonstrating platelet fibrinogen binding following stimulation with 10 µM ADP. C. Percentage of platelets binding fibrinogen at basal levels and in response to the platelet agonists ADP (0.1–10 µM) or TFLLR (1–10 µM). * P≤0.01.

The percentage of platelets binding fibrinogen was significantly higher in IPF patients compared with controls following stimulation with 0.1 µM and 1 µM ADP (0.1 µM ADP: 50.3%±8.9 versus 17.5%±6.1; p = <0.01; 1 µM ADP: 77.9%±4.6 versus 56.2%±6.5; p = <0.01). There was no significant difference in fibrinogen binding under basal (unstimulated) conditions (19.9%±6.0 versus 8.7%±3.2 respectively; p = 0.13). The increase in fibrinogen binding in IPF subjects following stimulation with TFLLR did not reach statistical significance (figure 4 and table S5 in file S1).

### The influence of the plasma environment on platelet function in IPF

**Figure 5:**
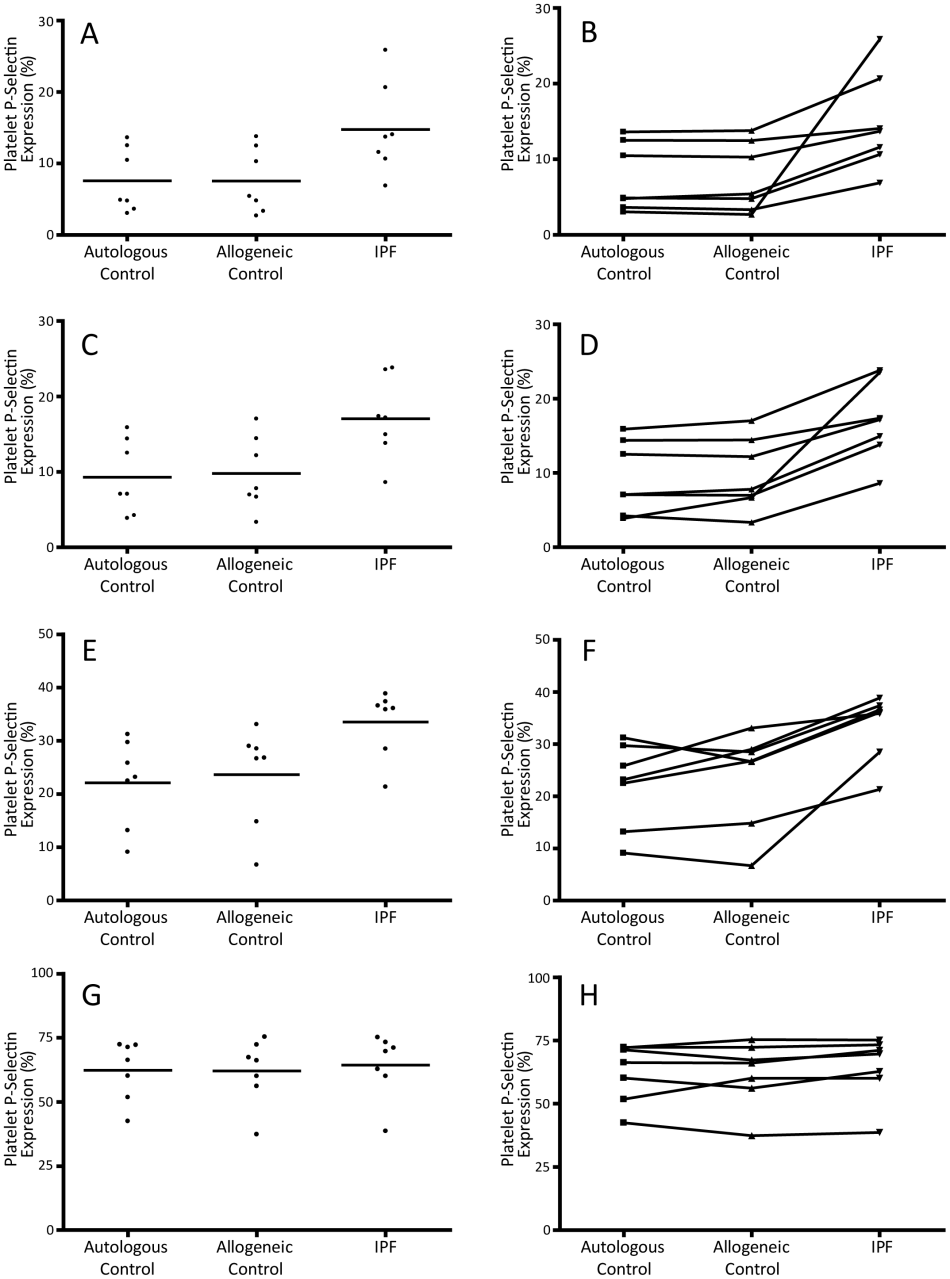
The influence of the plasma environment on platelet function in IPF patients. Scatter plots (A,C,E,G) demonstrating platelet P-selectin expression (bars indicate means) and line graphs (B,D,F,H) showing the change in P-selectin expression of control platelets following incubation in autologous plasma, allogeneic control plasma, and IPF patient plasma at basal levels (A,B) and following stimulation with ADP 0.1 µM (C,D), 1 µM (E,F) and 10 µM (G,H).

We next examined whether the platelet hyperactivity observed in IPF was caused by a primary platelet defect or the plasma environment. Re-suspension of washed control platelets in plasma from IPF subjects led to elevated P-selectin expression when compared with platelets suspended in autologous control or allogeneic control plasma. IPF plasma increased P-selectin expression under both basal conditions (p<0.05) and following stimulation with ADP at concentrations of 0.1 and 1 µM (P≤0.01) (figure 5 and table S6 in file S1). Further analysis was performed to confirm the increase in platelet P-selectin expression seen in IPF plasma. From a baseline taken as the platelet P-selectin expression in the autologous control group, the increase in platelet P-selectin expression in the presence of IPF plasma was significantly greater than the allogeneic control group at basal levels and following stimulation with all concentrations of ADP (p<0.05).

## Discussion

We have demonstrated platelet hyperactivity in patients with IPF using two mechanistically different agonists (ADP and TFLLR) and three different markers of platelet activation, namely platelet-monocyte aggregate formation, platelet P-selectin expression, and platelet fibrinogen binding. Increased platelet-monocyte aggregate formation is a sensitive marker of platelet activation [13] and demonstrates a functional consequence of the increased platelet reactivity. These data provide robust evidence of increased platelet reactivity in IPF.

To explore whether the hyperactivity phenotype in IPF was the result of an alteration of the platelets or their environment we used the plasma swap approach, which has been used successfully in previous studies to examine the role of plasma [14]. Incubation of washed control platelets in IPF plasma increased platelet activation as assessed by P-selectin expression both under basal conditions and after stimulation with ADP. Platelet hyperactivity was not observed when platelets were incubated with control plasma. This indicates that the plasma environment in IPF is responsible for the observed increased platelet reactivity. Further investigation is required to identify the plasma factor or factors responsible for this effect.

The two patient groups in this study were well matched in terms of age and gender. At the time of undertaking the platelet assays there was no evidence-based disease modifying treatment for IPF [2] and therefore it was local practice not to treat IPF patients with immunosuppressant drugs. This is reflected in our cohort with only 1 IPF patient receiving long term low dose prednisolone. One patient in the control group was also taking long term low dose prednisolone as treatment for COPD.

Although not statistically significant, there was a higher prevalence of cerebrovascular disease in the IPF group. There is a recognised association between cerebrovascular disease and platelet activation [15], but repeat analysis of our data following exclusion of patients with a history of cerebrovascular disease did not alter our conclusions. The platelet assays used in this study are not affected by aspirin [16] and reanalysis of the data following exclusion of patients using antiplatelet therapies did not alter our conclusions.

It has recently been demonstrated that patients with COPD have increased levels of circulating platelet-monocyte aggregates compared to age and smoking status matched controls [9]. The high prevalence of COPD in our control group may therefore result in the differences between the groups being underestimated, adding further support to the significance of the observed increased platelet reactivity in patients with IPF. The current smokers in this study were evenly distributed between IPF and control groups, indicating that the documented increased platelet activation and surface expression of P-selectin in smokers [17] will not have influenced our conclusions.

It is recognised that the choice of ex-vivo anticoagulant can impact platelet-monocyte association. Citrate results in lower levels of platelet-monocyte aggregates secondary to calcium chelation [18] whereas heparin-based anticoagulants may activate platelets through interactions with the platelet integrin α_IIb_β_3_ [19]. There is currently no consensus on the optimal anticoagulant for assessing platelet-monocyte aggregates. To minimise platelet activation we opted to use sodium citrate, which may have reduced our ability to detect a significant difference in platelet activation at basal levels in the present study. Since both groups were subject to the same conditions the differences observed between the groups will not have been affected by the choice of anticoagulant, but caution should be exercised when comparing our results with those from other studies using different anticoagulants.

The present study does not establish a temporal relationship between platelet activation and development of IPF, and in isolation we cannot conclude whether the observed increased platelet reactivity is an important phenomenon at the onset of the disease or represents a secondary response to pulmonary fibrosis. Although epidemiological studies have reported that the increased incidence of cardiovascular events and venous thromboembolism in IPF patients predates the presentation of lung disease [3], [4], caution must be exercised when drawing conclusions regarding cause and effect. However, it is important to recognise the pro-fibrotic potential of activated platelets. Platelet degranulation leads to the release of pro-fibrotic mediators including platelet derived growth factor (PDGF) [20] and transforming growth factor-beta (TGF-β) [21], which have been proposed to be key mediators in the pathogenesis of IPF [22]–[24]. We suggest that the increased platelet reactivity in patients with IPF may perpetuate the pulmonary fibrotic process through platelet sequestration in the lung and local release of pro-fibrotic mediators. Indeed, lung retention of platelets has been described in the bleomycin animal model of pulmonary fibrosis [10].

Therefore, in this small study of platelet activation in IPF we present clear evidence of increased platelet reactivity in IPF patients compared to controls and demonstrate that this effect can be reproduced in control platelets incubated in IPF plasma. This provides insight into the link between IPF and vascular diseases and we have described a mechanism by which platelet activation may perpetuate fibrosis. Activation of platelets represents an early stage in thrombosis and is unaffected by treatment with the vitamin K antagonist warfarin. The lack of benefit from anticoagulant therapies in IPF may be because the wrong component of the thrombotic process has been targeted [7].

## Conclusion

We have demonstrated increased platelet reactivity in IPF patients that is reproducible with diverse agonists and different markers of platelet activation. The increased activation and reactivity of control platelets when incubated in IPF plasma suggests that the abnormal platelet response in IPF is due to the plasma environment. This is the first report of increased platelet reactivity in IPF patients providing insight into the relationship between IPF and vascular disease and highlighting the need for further investigation of the role of platelets in IPF pathogenesis.

## Acknowledgments

We thank Dr Laura Sadofsky and Chris Crow for helpful comments and technical support and Dr Jakob Dudziak for his ICT expertise.

## Supporting Information

File S1 **Tables S1-S6.**
(DOCX)
