# Supplementary material for: Increased Platelet Reactivity in Idiopathic Pulmonary Fibrosis Is Mediated by a Plasma Factor
Source: PLoS One. 2014 Oct 22;9(10):e111347. doi: 10.1371/journal.pone.0111347 (PMC4206466; doi:10.1371/journal.pone.0111347)
Supplement: File S1 — Tables S1-S6. (DOCX) [file pone.0111347.s001.docx]

**Title:** Increased platelet reactivity in idiopathic pulmonary fibrosis is mediated by a plasma factor

**Dr Michael G. Crooks^1^**, **Dr Ahmed Fahim^2^, Professor Khalid Naseem^1^, Professor Alyn H. Morice^1^, Dr Simon P. Hart^1^**

**Data Supplement File S1**

**Definition of Smoking Status**

For the purposes of this study current smokers were classified as those who had smoked in the preceding 6 months, ex-smokers as those who had stopped smoking greater than 6 months before sampling, and non-smokers as those who had never smoked.

**Plasma swap Protocol**

Washed platelets suspended in PBS were separated into three 100μl aliquots. 100μl of autologous plasma was added to the first aliquot of washed platelets, 100μl of plasma from a control patient was added to the second alliquot and 100μl of plasma from an IPF patient was added to the final aliquot resulting in 3 suspensions of washed platelets constituting ‘autologous control’, ‘allogeneic control’ and ‘IPF’ samples respectively. 5μl of each sample was individually incubated for 20 minutes in basal conditions and in the presence of ADP (0.1, 1 and 10μM) with PE-conjugated anti-human CD62P (Biolegend, San Diego, California). A separate sample was incubated with FITC-conjugated anti CD42b to confirm that the gated population represented platelets. The samples were made up to 50μl with PBS. Following incubation samples were fixed with 500μl of 1% paraformaldehyde prior to flow cytometry.

| **Demographic** | **IPF (%)** | **Controls (%)** | **p-value** |
| --- | --- | --- | --- |
| **Number** | **n=13** | **n=12** |  |
| **Age (mean)** | **70.3** | **66.2** | 0.22 |
| <50 | 0 (0) | 0 (0) | - |
| 51-60 | 0 (0) | 4 (33.3) | - |
| 61-70 | 8 (61.5) | 3 (25) | - |
| 71-80 | 5 (38.5) | 4 (33.3) | - |
| >80 | 0 (0) | 1 (8.3) | - |
| **Gender** |  |  |  |
| Male | 9 (69.2) | 8 (66.7) | 1.00 |
| Female | 4 (30.8) | 4 (33.3) | 1.00 |
| **Comorbidities** |  |  |  |
| COPD | 2 (15.4) | 6 (50) | 0.09 |
| Prev. malignancy | 2 (15.4) | 1 (8.3) | 1.00 |
| Hypertension | 4 (30.8) | 1 (8.3) | 0.30 |
| Diabetes mellitus | 0 (0) | 2 (16.7) | 0.22 |
| TIA | 3 (23.1) | 0 (0) | 0.22 |
| Stroke | 0 (0) | 0 (0) | 1.00 |
| Atrial Fibrillation | 1 (7.7) | 2 (16.7) | 0.58 |
| **Anti-platelet drug use** |  |  |  |
| Aspirin | 4 (30.8) | 0 (0) | 0.09 |
| Clopidogrel | 0 (0) | 0 (0) | 1.00 |
| Dipyridamole | 1 (7.7) | 0 (0) | 1.00 |
| **Smoking status** |  |  |  |
| Non-smoker | 1 (7.7) | 6 (50) | 0.03 |
| Ex-smoker | 10 (77.9) | 4 (33.3) | 0.04 |
| Current smoker | 2 (15.4) | 2 (16.7) | 1.00 |

**Table S1.** Assessment of platelet activation: baseline characteristics of IPF patients and controls

| **Demographic** | **IPF (%)** | **Controls (%)** | **p-value** |
| --- | --- | --- | --- |
| **Number (n)** | 7 | 7 |  |
| **Age in years – Mean (SD)** | 74 (8) | 65 (10) | 0.07 |
| **Gender (%)** |  |  |  |
| Male | 6 | 4 | 0.56 |
| Female | 1 | 3 | 0.56 |
| **Comorbidities (%)** |  |  |  |
| IHD | 1 | 0 | 1.00 |
| TIA/CVA | 1 | 0 | 1.00 |
| Hypertension | 1 | 2 | 1.00 |
| Diabetes mellitus | 1 | 0 | 1.00 |
| GORD | 2 | 2 | 1.00 |
| COPD | 0 | 2 | 0.46 |
| Sleep apnoea | 0 | 2 | 0.46 |
| **Smoking Status (%)** |  |  |  |
| Current | 0 | 0 | 1.00 |
| Ex-smoker | 4 | 4 | 1.00 |
| Non-smoker | 2 | 2 | 1.00 |
| Unknown | 1 | 1 | 1.00 |

**Table S2.** Assessment the effect of IPF plasma on control platelets: baseline characteristics of IPF patients and controls

| **Platelet Agonist** | **IPF (n=13)** | **Controls (n=12)** | **p-value** |
| --- | --- | --- | --- |
| **Basal** | **18.19 (3.88)** | **13.71 (1.35)** | **0.30** |
|  |  |  |  |
| **0.1µM ADP** | **24.08 (4.68)** | **15.28 (1.49)** | **0.09** |
| **1µM ADP** | **29.40 (4.12)** | **16.10 (1.52)** | **<0.01** |
| **10µM ADP** | **44.83 (3.28)** | **32.11 (3.49)** | **0.01** |
|  |  |  |  |
| **1µM TFLLR** | **28.59 (4.54)** | **18.65 (1.79)** | **0.06** |
| **5µM TFLLR** | **41.73 (2.47)** | **27.03 (3.44)** | **<0.01** |
| **10µM TFLLR** | **61.05 (3.93)** | **41.09 (3.5)** | **<0.01** |

**Table S3.** Mean (SEM) percentage of monocytes with one or more bound platelets (platelet-monocyte aggregates) in IPF and controls at basal levels and in response to the platelet agonists ADP and TFLLR.

| **Platelet Agonist** | **IPF (n=13)** | **Controls (n=12)** | **p-value** |
| --- | --- | --- | --- |
| **Basal** | **0.99 (0.14)** | **0.70 (0.18)** | **0.25** |
|  |  |  |  |
| **0.1µM ADP** | **1.86 (0.46)** | **0.72 (0.13)** | **0.03** |
| **1µM ADP** | **9.78 (1.25)** | **3.25 (0.77)** | **<0.01** |
| **10µM ADP** | **41.27 (4.2)** | **22.52 (2.64)** | **<0.01** |
|  |  |  |  |
| **1µM TFLLR** | **3.05 (1.66)** | **0.99 (0.25)** | **0.25** |
| **5µM TFLLR** | **16.82 (5.29)** | **5.28 (2.06)** | **0.06** |
| **10µM TFLLR** | **51.53 (9.24)** | **23.10 (5.48)** | **0.02** |

**Table S4.** Mean (SEM) percentage of platelets expressing P-selectin under basal conditions and following stimulation with the platelet agonists ADP and TFLLR.

| **Platelet Agonist** | **IPF (n=13)** | **Controls (n=12)** | **p-value** |
| --- | --- | --- | --- |
| **Basal** | **19.92 (5.96)** | **8.67 (3.19)** | **0.13** |
|  |  |  |  |
| **0.1µM ADP** | **50.28 (8.85)** | **17.48 (6.13)** | **<0.01** |
| **1µM ADP** | **77.92 (4.63)** | **56.24 (6.53)** | **<0.01** |
| **10µM ADP** | **92.13 (1.69)** | **86.36 (2.54)** | **0.07** |
|  |  |  |  |
| **1µM TFLLR** | **49.30 (8.94)** | **31.82 (7.0)** | **0.15** |
| **5µM TFLLR** | **68.49 (7.71)** | **46.61 (7.6)** | **0.06** |
| **10µM TFLLR** | **83.10 (5.3)** | **71.78 (6.25)** | **0.18** |

**Table S5.** Mean (SEM) percentage of platelets with bound fibrinogen in basal conditions and following stimulation with the platelet agonists ADP and TFLLR.

| **Platelet Agonist** | **Autologous Control** | **Allogeneic Control** | **IPF** |
| --- | --- | --- | --- |
| **Basal** | **7.56 (1.69)** | **7.54 (1.72)** | **14.78 (2.44)** |
|  |  |  |  |
| **0.1µM ADP** | **9.29 (1.86)** | **9.78 (1.84)** | **17.04 (2.04)** |
| **1µM ADP** | **22.1 (3.11)** | **23.64 (3.54)** | **33.51 (2.39)** |
| **10µM ADP** | **62.36 (4.38)** | **62.09 (4.82)** | **64.39 (4.76)** |

**Table S6.** Mean (SEM) percentage of platelets expressing P-selectin following incubation in autologous plasma (Autologous Control), control patient plasma (Allogeneic Control) and IPF plasma (n=7).
